# Magnitude of institutional delivery service utilization and associated factors among women in pastoral community of Awash Fentale district Afar Regional State, Ethiopia

**DOI:** 10.1186/s13104-018-3261-5

**Published:** 2018-03-02

**Authors:** Luelseged Assefa, Mussie Alemayehu, Ayal Debie

**Affiliations:** 1 Afar Regional Health Bureau, Afar National Regional State, Samara, Ethiopia; 2 Department of Reproductive Health, Mekelle University, Mekelle, Ethiopia; 3 Department of Health Service Management and Health Economics, University of Gondar, P.o.Box: 196, Gondar, Ethiopia

**Keywords:** Institutional delivery, Awash Fentale district, Pastoral community, Afar region

## Abstract

**Objective:**

Reduction of maternal mortality is a global priority particularly in developing countries like Ethiopia where maternal mortality ratio is one of the highest in the world. Most deliveries in developing countries occur at home without skilled birth attendants. Therefore, the objective of this study was to assess institutional delivery service utilization and associated factors among women in pastoral community of Awash Fentale district, Ethiopia.

**Results:**

Overall, 35.2% of women delivered at health facilities. Women who had good knowledge AOR = 2.1, 95% CI 1.32, 4.87), Ante Natal Care (ANC) follow up (AOR = 3.2, 95% CI 1.55, 6.63), resided in a place where distance to reach at the nearby health facilities takes < 30 min (AOR = 3.1; 95% CI 2.57, 66.33) and women whose husband involved in decision regarding delivery place (AOR = 1.9; 95% CI 1.49, 5.07) were more likely to deliver at health facility. Therefore, strengthening ANC services, improving maternal knowledge, involving husbands in decision of delivery place and expanding health facilities in the community would enhance institutional delivery.

**Electronic supplementary material:**

The online version of this article (10.1186/s13104-018-3261-5) contains supplementary material, which is available to authorized users.

## Introduction

Delivery attended by qualified health care providers is the best strategy in reducing maternal mortality in the world and one of the indicators to track national effort towards safe motherhood [1]. Maternal mortality ratio in Ethiopia is among the highest in sub-Saharan Africa which was 676 per 100,000 live births as a result of low maternal service utilization particularly low institutional delivery [2]. Skilled delivery is low in southern Asia and sub-Saharan Africa (SSA) which is 40 and 47%, respectively [3]. Institutional delivery is also low in Ethiopia ranged from 4.1 to 18.2% of women gave birth at health facilities [4–6]. In Afar region, 93.6% of mothers give birth at home which is one of the highest home delivery practices among the nine regions of Ethiopia [7].

Institutional delivery ensures safe birth, reduces occurrence of complication during delivery and immediately after birth and increases the survival of mothers and newborns [8, 9]. However, majority of deliveries in Ethiopia particularly in pastoral community of Afar region occur at home without skilled birth attendants. This is because; in the region nomads are moving seasonally from place to place for the purpose of feeding and getting water for their cattle. Hence mothers may not have access to health facilities in the nearby [7, 10, 11]. Therefore, recognizing factors affecting institutional delivery service utilization in pastoral community has paramount importance in order to design a strategy for enhancing institutional delivery service utilization. Accordingly, this study was intended to assess magnitude of institutional delivery service utilization and associated factors among reproductive age women in pastoral community of Awash Fentale district, Ethiopia.

## Main text

### Methods

A community based cross-sectional study was employed among women of childbearing age group (15–49 years) in Pastoral community of Awash Fentale district who gave birth in the last 1 year preceding the study. The district has 5 rural kebeles with a total population of 29,076 within 5101 households. The total number of reproductive age women (15–49 years) also estimated to be 4035 [12]. The study was conducted from February to May, 2016. The source and study populations were all women with a child of less than 12 months old from all rural kebeles of the district. All mentally capable women (conscious and free from a known psychiatric disorder) with a child less than 12 months and resided in the respective kebeles at least for the past 6 months were included in the study while mothers who were unable to communicate and seriously ill were excluded from the study.

Sample size was calculated by using both single and double population proportion formula. Sample size for first objective was calculated by using single population proportion formula with an assumption: Z*a*_*/2*_ at 95% confidence level = ± 1.96, margin of error (w) = 3%, and proportion (P) = 6.4% [7], design effect = 1.5 and non response rate = 10%. N = (Z*a*_*/2*_)^2^(P)(1 − P)/(*w)*^2^ = (1.96)^2^ (0.064) (0.936)/(0.03)^2^ = 256; after adding 10% non response rate it became 282. The final sample size for this objective after multiplying by design effect 1.5 was 423. Sample size for second objective was calculated by using double population proportion formula by using significantly associated variables from the previous study with an assumption of power = 80%, 95% level of confidence, design effect = 1.5. The identified variables were history of ANC follow up, mothers’ residence, mothers’ educational status and wealth status [11, 13, 14]. The final sample sizes for this objective based on these factors were 96, 53, 376, and 53, respectively. Using the largest sample size is more appropriate for maintaining sample size adequacy. Therefore, the final sample size was decided to be 423. During selection of study participants, first the sample size was proportionally allocated to each selected kebeles and finally, cluster sampling technique was used to select mothers.

The dependent variable of the study was institutional delivery service utilization. The independent variables were socio-demographic characteristics (age, religion, level of education, marital status, mothers and husband occupation, and level of income); previous obstetric history of mothers (gravidity, parity, abortion and still birth/intrauterine fetal death); health seeking related factors (women’s knowledge, attitude, perception, decision making power on place of delivery), and accessibility related factors (transportation and time spent to reach at health facility). The knowledge of mothers on pregnancy and labor was measured by using 9 questions and those mothers who scored 50% and above of the knowledge related questions were considered to have good knowledge and those mothers scored below 50% of the knowledge related questions were considered to have poor knowledge. Whereas, the attitude of mothers towards institutional delivery was measured by using 9 questions and those mothers who scored on 50% and above of the attitude related questions were considered to have favorable attitude and those mothers scored below 50% of the attitude measurement questions were considered to have unfavorable attitude.

A structured questionnaire, adopted from different literatures, was used for data collection [7, 11, 15, 16]. The questionnaire was first prepared in English and then, translated to the local language. Finally, it was back to English in order to ensure consistency. The data collectors and supervisors were trained for 2 days mainly on the objectives of the study and on the basic techniques of the interviewing process. The questionnaire was pre-tested on 21 child bearing age women in the rural kebele of Kurkura in Amibara district. Unclear, misunderstood or ambiguous words or questions were modified based on the pretest findings. Supervisors and the principal investigators checked the completeness and consistency of the questionnaire on daily bases in order to ensure the data quality. The collected data were checked for completeness and consistency and entered into EPI info version 3.5.3 and exported to SPSS version 20 for analysis. Descriptive statistics such as frequency and percentage were presented by using tables and graphs. Logistic regression analysis was carried out to identify factors associated with institutional delivery service utilization. Those independent variables having P value less than 0.2 during bivariate analysis were included in the multivariable analysis. Crude and adjusted Odds ratios (OR) with 95% CI and P value < 0.05 were used to identify the strength and level of significance of the association.

## Results

### Socio-demographic characteristics of women

A total of 423 mothers participated in the study with a response rate of 100%. The mean age of respondents was 28.84 ± 6.55 years, and the median age of women was 29 years. From all study participants, 50% of the respondents were Afar in their ethnicity, 94.6% were currently married and 60.8% were Muslim religion followers. From all currently married mothers, 68% of the respondents’ husbands were unable to read and write and 60% of mothers were unable to read and write. Out of the total participants, 12.8% were housewife, and 62% of their husbands’ were pastoralist. 47.8% of respondents their monthly household income was in the range of 500–1000 Ethiopian Birr (ETB) (Table 1).

**Table 1:** Socio-demographic characteristics of respondents in Awash Fentale district, Afar region, 2016 (N = 423)

Characteristics	Frequency	Percentage (%)
Age of women (n = 423)		
15–19	61	14.4
20–24	54	12.8
25–29	110	26.0
30–34	107	25.3
35–39	63	14.9
≥ 40	32	7.6
Religion (n = 423)		
Muslim	257	60.8
Christian	166	39.2
Ethnicity (n = 423)		
Afar	211	49.9
Oromo	45	10.6
Tigray	16	3.8
Amhara	66	15.6
Others	85	20.1
Women occupation (n = 423)		
House wife	54	12.8
Farmer	75	17.7
Employed	10	2.4
Merchant	33	7.8
Pastoralist	251	59
Husband occupation(n = 413)		
Pastoralist	257	62
Farmer	82	19.9
Employed	43	10.4
Daily labor	20	5
Merchant	11	2.7
Monthly household income ETB (n = 423) (birr)		
< 500	36	8.5
500–1000	202	47.8
> 1000	185	43.7
Maternal educational status (n = 423)		
Unable to read and write	252	60
Primary school	121	29
Secondary and above	50	12
Husband educational status(n = 413)		
Unable to read and write	281	68
Primary school	68	16.1
Secondary school and above	67	16.9

### Obstetrics characteristics of women

Among the study participants, 76.4% of mothers got married before the age of 20 years and 67.6% who gave the recent birth were in age range of 20–34 years. From all respondents, 77.3% of women attended Ante Natal Care (ANC) during the last pregnancy. Out of all mothers, 64.5% of them had two to three live children, 2.8% had history of intra uterine fetal death (IUFD) or still birth and 6.6% had history of abortion. 45.2% of study participants were given Tetanus Toxoid (TT) vaccination twice before birth and 76.1% of mothers faced obstetric complication during their previous delivery (Table 2, Additional file 1: Figure S1; Additional file 2: Figure S2).

**Table 2:** Obstetric characteristics of respondents in pastoral Awash Fentale district, Afar region, 2016 (N = 423)

Characteristics	Frequency	Percentage (%)
Age at first marriage (n = 423) (years)		
< 20	323	76.4
≥ 20	100	23.6
Women’s age during recent birth (n = 423) (years)		
< 20	42	9.9
20–34	286	67.6
35–49	95	22.5
Gravidity (n = 423)		
One	63	14.9
Two to three	115	27.2
Four to five	121	28.6
≥ Six	124	29.2
Parity (n = 423)		
One	66	15.6
Two–three	116	27.4
Four to five	118	27.9
≥ Six	123	29
ANC follow up during last pregnancy (n = 423)		
Yes	327	77.3
No	96	22.7
Number of child alive (n = 423)		
One	3	0.7
Two to three	273	64.5
≥ Four	147	34.8
History of IUFD/still birth (n = 423)		
No	411	97.2
Yes	12	2.8
History of abortion (n = 423)		
No	395	93.4
Yes	28	6.6
Tetanus toxoid vaccination before birth (n = 423)		
Not at all	74	17.5
Once	23	5.5
Twice	191	45.2
Three and more	135	32
Obstetric complication during previous delivery (n = 423)		
Yes	322	76.1
No	101	23.9

### Factors associated with institutional delivery service utilization

Mothers who had good knowledge on institutional delivery service utilization were 2.1 times (AOR = 2.1; 95% CI 1.32, 4.87) more likely to use institutional delivery service as compared to those women having poor knowledge.

Women who were attending Antenatal Care follow up during last pregnancy were 3.2 times (AOR = 3.2; 95% CI 1.55, 6.63) more likely to deliver at health facilities than women who did not attend ANC.

Women who had to travel 30 min to reach at the nearby health facilities were 3.1 times (AOR = 3.1; 95% CI 2.57, 66.33) more likely to deliver at health facility as compared to those women who had to travel more than 30 min to reach to the nearby health facilities.

Women whose husband involved in decision regarding place of delivery were 1.9 times (AOR = 1.9; 95% CI 1.49, 5.07) more likely to deliver at health facility as compared to women whose husband did not involve in the decision making process of their delivery place (Table 3).

**Table 3:** Factors associated with institutional delivery service utilization among women in pastoral Awash Fentale district, 2016

Variables	Institutional delivery		COR	AOR
	Yes	No	(95% CI)	(95% CI)
Age of respondent				
15–19	17	44	1	1
20–34	85	182	1.2 (0.65, 2.24)	1.1 (0.53, 2.33)
≥ 35	47	48	2.5 (1.27, 5.05)	2.1 (0.88, 4.85)
ANC follow up				
Yes	134	193	3.7 (2.07, 6.79)	3.2 (1.55, 6.63)*
No	15	81	1	1
Distance to health facility (min)				
≤ 30	126	213	1.6 (0.83, 50.95)	3.1 (2.57, 66.33)*
> 30	23	61	1	1
Attitude of women				
Favorable	137	214	3.2 (0.12, 12.14)	1.2 (0.28, 2.98)
Unfavorable	12	60	1	1
Knowledge of women				
Good	75	28	8.8 (1.58, 13.32)	2.1 (1.32, 4.87)*
Poor	74	244	1	1
Husband involved on decision of delivery place				
Yes	62	80	1.7 (0.91, 2.86)	1.9 (1.49, 5.07)*
No	87	194	1	1

* Significant at P value < 0.05

## Discussion

The magnitude of institutional delivery service utilization in the study area was 35.2%. However, the remaining 64.8% of women gave birth at their homes. This study was comparable with a study conducted in Ghana (37.5%) [17], and it is lower than the studies conducted in other parts of Ethiopia such as Bahir Dar (78.8%) [18], Woldia (48.3%) [19], Tigray (59%), Diredawa (59%), Addis Ababa (79%) [2], Tigray (57%) and Addis Ababa (97%) [20]; and Tanzania (74.5%) [21]. However, it is higher than findings of the studies conducted in other parts of Ethiopia such as Munisa (12.3%) [13], Sekela (12.1%) [22], Dodota (18.2%) [5], rural Jimma Horro (8%) [23], Banja (15.7%) [24] and mini-EDHS 2014 report of Ethiopia (16%) [7], EDHS 2011 report of Ethiopia (10%) [2] and EDHS 2016 report of Ethiopia (26%) [10]. This might be due to difference in study area, period, and study participants. Difference in health facilities’ infrastructure might affect mothers’ institutional delivery service utilization. Health facilities which had maternal waiting home and presence of ambulance service might also give a chance for women to attend institutional delivery.

In this study, those mothers who had history of ANC follow up were 3.2 times more likely (AOR = 3.2; 95% CI 1.55, 6.63) attend institutional delivery than mothers who had no history of ANC follow up. This finding was supported by a study conducted in Munisa, Sekela and Western Ethiopia [13, 22, 26]; Tanzania [21], Ghana [17], and Nigeria [25]. The possible explanation might be mothers who decide to attend institutional delivery as a result of the information they got during their prenatal care.

Women who had to travel 30 min to reach at the nearby health facilities were 3.1 times (AOR = 3.1; 95% CI 2.57, 66.33) more likely to deliver at health facility as compared to those women who had to travel more than 30 min to reach to the nearby health facilities. This finding was consistent with the study conducted from Western Ethiopia and Dembecha district [26, 27]. This might be due to mothers resided to the nearby health institutions might have different access of maternal health services such as health education and ANC services. Moreover, mothers who resided to the nearby health facilities had no problem of transportation to attend institutional delivery at any time. In addition, in pastoral community most of husbands and adult male family members were seasonally moving from place to place to fed and get water for their cattle and it results mothers couldn’t get any support to take them to health facilities during the time of labor.

Those women who had good knowledge about attending health facility delivery were 2.1 times more likely (AOR = 2.1; 95% CI 1.32, 4.87) to deliver at health facilities when compared with women who had poor knowledge. This finding was in line with the study conducted at Sekela district and Western Ethiopia [22, 26]. This might be due to those women who had good knowledge might have high attitudinal change towards institutional delivery. This may help pregnant mothers to predict bad delivery outcomes. The other justification might be mothers having good knowledge may not be relatively affected by traditional malpractices and/or beliefs such as they might not consider mothers delivered at home as a hero of the community. Furthermore, knowledgeable mothers might be highly influential on their husbands and/or other relatives to take them to health facility while they were in labor.

Women whose husband involved in decision of delivery place were 1.9 times more likely (AOR = 1.9; 95% CI 1.49, 5.07) deliver at health facility than women’s husband who did not involve. This study finding was consistent with the study conducted at Dodota district [5].This might be due to male dominancy was common in pastoral community. However, husbands involved in maternal obstetric services during their pregnancy would decide in better way to attend their wives’ childbirth at health facility. Generally, this study finding revealed that majority of mothers still delivered at home. Therefore, strengthening ANC services, improving maternal knowledge, involving husbands in decision regarding delivery place and expanding health facilities to the nearby the community would enhance institutional delivery. Ministry of Health (MOH) and Regional Health Bureaus had better to use this study finding for policy making in the case of pastoral community throughout the country.

### Limitations

- Mothers might lead to a recall bias due to data was collected from mothers about their experience since 1 year back and.
- The study could not allow cause effect relationship because of its cross sectional nature.

## Additional files

**Additional file 1: Figure S1.** Knowledge, attitude, perception, husband involvement and distance towards institutional delivery in pastoral Awash Fentale district, 2016. Knowledge, attitude, perception, husband involvement, and distance to health facility. Among study participants, 24.3% of women had good knowledge on labor and pregnancy, and 83% of women had favorable attitudes towards institutional delivery. Moreover, 60.3% of women had good perception on the benefit of skilled birth attendance and 33.6% of women’s husband involved on decision regarding delivery place. Concerning the time taken, 19.9% of mothers travelled on foot more than 30 min to reach the nearby health facilities.

**Additional file 2: Figure S2.** Place of delivery in the last pregnancy among women in pastoral Awash Fentale district, Ethiopia, 2016. Prevalence of institution delivery service utilization. Among mothers who gave birth in the last 12 months, 35.2% of them delivered in health facilities while the rest 64.8% gave birth at home.

## Abbreviations

ARHB: Afar Regional Health Bureau
ANC: Ante Natal Care
EMDHS: Ethiopia Mini Demographic Health Survey
HD: Home Delivery
HIV: Human Immune Virus
IMR: Infant Mortality Rate
IUFD: Intra Uterine Fetal Death
ID: Institutional Delivery
MMR: Maternal Morality Ratio
PMR: Perinatal Mortality Rate
TBAs: Traditional Birth Attendants
WHO: World Health Organization
