# Supplementary figures and images for: Magnitude of institutional delivery service utilization and associated factors among women in pastoral community of Awash Fentale district Afar Regional State, Ethiopia
Source: BMC Res Notes. 2018 Mar 2;11:162. doi: 10.1186/s13104-018-3261-5 (PMC5833063; doi:10.1186/s13104-018-3261-5)

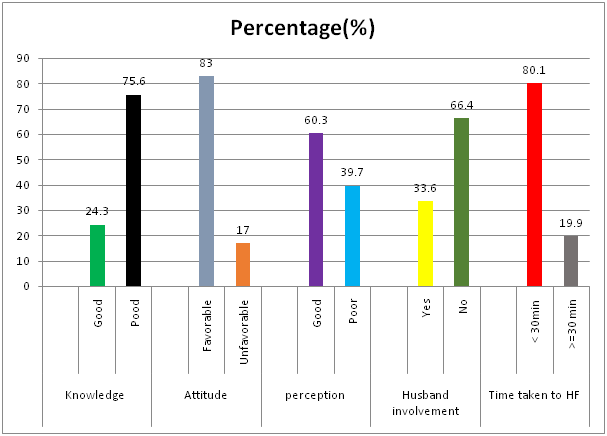

Supplement: Supplementary file 1 — Additional file 1: Figure S1. Knowledge, attitude, perception, husband involvement and distance towards institutional delivery in pastoral Awash Fentale district, 2016. Knowledge, attitude, perception, husband involvement, and distance to health facility. Among study participants, 24.3% of women had good knowledge on labor and pregnancy, and 83% of women had favorable attitudes towards institutional delivery. Moreover, 60.3% of women had good perception on the benefit of skilled birth attendance and 33.6% of women’s husband involved on decision regarding delivery place. Concerning the time taken, 19.9% of mothers travelled on foot more than 30 min to reach the nearby health facilities. [file 13104_2018_3261_MOESM1_ESM.tif]

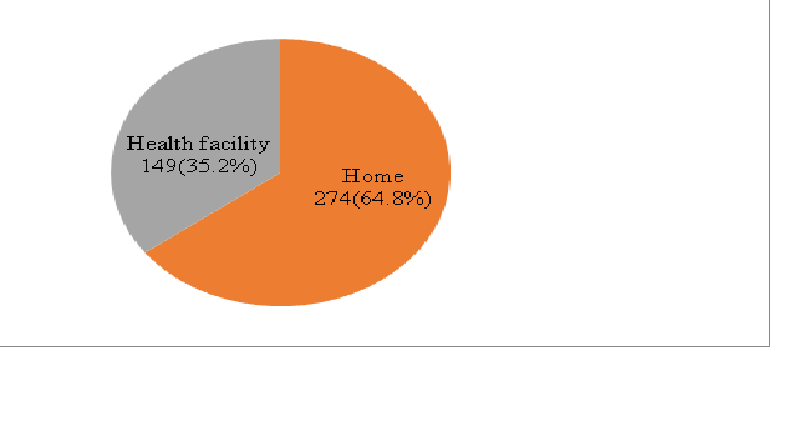

Supplement: Supplementary file 2 — Additional file 2: Figure S2. Place of delivery in the last pregnancy among women in pastoral Awash Fentale district, Ethiopia, 2016. Prevalence of institution delivery service utilization. Among mothers who gave birth in the last 12 months, 35.2% of them delivered in health facilities while the rest 64.8% gave birth at home. [file 13104_2018_3261_MOESM2_ESM.tif]
